# The COVID-19 Crisis as a Teachable Moment for Lifestyle Change in Dutch Cardiovascular Disease Patients

**DOI:** 10.3389/fpsyg.2021.678513

**Published:** 2021-06-22

**Authors:** Michelle Brust, Winifred A. Gebhardt, Mattijs E. Numans, Jessica C. Kiefte-de Jong

**Affiliations:** ^1^Department of Public Health and Primary Care/LUMC Campus The Hague, Leiden University Medical Center, The Hague, Netherlands; ^2^Department of Health, Medical and Neuropsychology, Leiden University, Leiden, Netherlands

**Keywords:** COVID-19, lifestyle, behavior change, cardiovascular disease, prevention

## Abstract

**Objective:** When lifestyle changes are needed, life events or crises such as COVID-19 may function as “teachable moments”. This study aimed to explore whether the pandemic can provoke a teachable moment regarding lifestyle change in cardiovascular disease patients.

**Method:** In this cross-sectional survey study, 830 cardiovascular disease patients reported their intentions to change lifestyle, instigated by the corona crisis, together with risk perception, affective impact, and changed self-concept, based on a “teachable moments” framework.

**Results:** Between 8 and 28% of the sample reported increased intentions to optimize lifestyle behaviors, particularly related to general lifestyle (28%), physical activity (25%), and diet (21%). Multivariate regression analyses revealed that changed self-concept was associated with higher intentions to improve general lifestyle (*B* = 0.26; CI = 0.19–0.33), physical activity (*B* = 0.23; CI = 0.16–0.30), and smoking (*B* = 0.29; CI = 0.01–0.57). In addition, changed self-concept and affective impact were both significantly associated with higher intentions to improve diet (resp. *B* = 0.29; CI = 0.21–0.36 and *B* = 0.12; CI = 0.04–0.21) and to limit alcohol consumption (resp. *B* = 0.22; CI = 0.13–0.30 and *B* = 0.11; CI = 0.01–0.20). We did not find evidence for an important role of risk perception on behavior change intentions.

**Conclusion:** The COVID-19 crisis evoked a potential teachable moment for lifestyle change in cardiovascular disease patients, driven by a change in a patient's self-concept and to a lesser extent by an affective impact of the COVID-19 crisis. These results suggest an important window of opportunity for healthcare professionals to utilize the pandemic to promote a healthy lifestyle to their patients.

## Introduction

The novel coronavirus (COVID-19) outbreak has rapidly emerged as a global health threat in a very short time frame: the first reported case of this respiratory infectious disease was dated in November 2019 and a pandemic was officially declared by the World Health Organization (WHO) in March 2020 (World Health Organization, 2020a). The number of individuals infected with SARS-CoV-2 has increased rapidly, leading to feelings of uncertainty, worry, and fear among large swaths of the population (Dubey et al., 2020). Evidence is accumulating to support the notion that being a non-smoker, having a healthy nutrition pattern, and engaging in regular physical activity serve as protective factors for adverse health consequences of COVID-19 (De Frel et al., 2020; Mattioli et al., 2020; Peçanha et al., 2020). Conversely, obesity and smoking are associated with a greater risk of developing severe COVID-19 outcomes, hospitalization, and death (De Frel et al., 2020; Vardavas and Nikitara, 2020). These insights suggest the COVID-19 crisis has the potential to be a prompting situation that raises awareness on the importance of adopting a healthier lifestyle, especially for individuals with a history of cardiovascular diseases (CVD), as they are a particularly vulnerable subpopulation with a higher risk of mortality and morbidity during an infection (Guzik et al., 2020; Madjid et al., 2020).

Situations that urge individuals to start living a healthier life are known as “teachable moments” (TMs) (McBride et al., 2003; Lawson and Flocke, 2009). TMs generally arise during or after significant life or health events such as hospitalization, pregnancy, or severe disease diagnosis, any of which may cause an individual to become more receptive to health behavior messages and encourage changes in health behaviors (McBride et al., 2003, 2017; Cohen et al., 2011; Okely et al., 2019). According to a conceptual framework proposed by McBride et al. (2003), three facilitating characteristics allow life events to serve as effective TMs in terms of instigating health behavior change. First, an event should be characterized by an increase in risk perception. Second, an event must prompt a strong affective or emotional response for it to be perceived as significant and meaningful enough to prompt behavior change. And third, an event must lead to a change in a person's self-concept, in order to evoke the adoption of healthier behaviors. Tofler et al. (2015) found evidence for a TM to stop smoking after patients were hospitalized with acute coronary syndrome. One year after hospitalization, the abstinence rate of their study participants remained as high as 61%. Further research is needed to establish whether TMs cause permanent behavior changes in CVD patients or whether new events are necessary to re-activate motivation. Given its novelty, little is known about whether the COVID-19 crisis can function as such a TM.

Although the pandemic may increase willingness toward healthier behaviors, home isolation and psychological strain may also induce unhealthy behaviors such as sedentary behavior, smoking, an unhealthy diet, and excessive alcohol consumption (Block et al., 2009; Slopen et al., 2013; Lippi et al., 2020). This lifestyle pattern is not only associated with increased mortality and morbidity risk following COVID-19, but may also lead to a worsening of cardiometabolic outcomes (Mattioli et al., 2020; Peçanha et al., 2020). With 1.7 million Dutch CVD patients in 2019, cardiometabolic disorders are the most common type of chronic diseases in the Netherlands, and even the leading cause of death globally (World Health Organization, 2020b). Because of this high prevalence, it is very important and relevant to understand how the COVID-19 crisis influences motivation for a healthy lifestyle among CVD patients.

**Figure 1:**
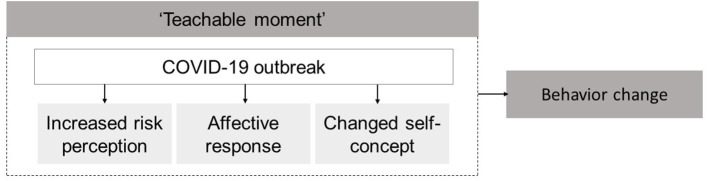
Conceptual framework based on McBride et al. (2003).

The aim of our study is to investigate whether the COVID-19 crisis has provoked a TM in Dutch CVD patients. We also explore whether a TM, if it occurs, is related to perceived risk perception, affective or emotional impact, and change in self-concept resulting from the COVID-19 crisis (Figure 1). These are key factors derived from the TM framework proposed by McBride et al. (2003), but have never been empirically tested in relation to the COVID-19 crisis. Insights into these facilitating factors that turn the COVID-19 crisis into a TM could inform health promotion approaches during the development of tailored (online) lifestyle counseling interventions, and may as such further the optimization of lifestyle behaviors of CVD patients during or after the pandemic. We hypothesized that the COVID-19 crisis would lead to a TM, increasing both motivation to improve lifestyle behaviors and awareness of the importance of a healthy lifestyle.

## Method

### Study Design and Participants

For this cross-sectional survey study, we used the online survey software program Qualtrics (www.Qualtrics.com). Recruitment and data collection took place during a 14-day window from May 5th, 2020 through May 19th, 2020. Individuals who were 18 years or older with a history of cardiometabolic diseases were eligible to participate in the study. Data collection was completely anonymous. The majority of participants were recruited via Harteraad, the national Dutch patient association for individuals living with CVD. This patient association comprised at the time of data collection a total of 2,606 CVD patients with diverse sociodemographic characteristics. All members of the patient association were invited to voluntarily participate in the study through an e-mail that was sent by the organization of Harteraad. In addition, participants were recruited via the first authors' personal social media (Facebook) channel. We confirmed whether participants were eligible to participate on the first page of the questionnaire, where they could specify their history of CVD. We chose these recruitment methods to approach as large a cohort as possible throughout the country, to obtain a broad insight into behavior change intentions during the COVID-19 crisis. By approaching members of the largest national patient association for individuals living with CVD, we aimed to recruit a representative sample of the general Dutch CVD population. All participants could access the online questionnaire by clicking on an anonymous link in the e-mail and social media post. After providing online informed consent on the first page of the questionnaire, participants could start the survey. The Medical Ethics Committee of Leiden University Medical Center approved and registered the study (METC-nr 18-112).

### Measures

Intentions to optimize lifestyle as a result of the COVID-19 crisis were assessed using subscales from a larger survey, developed and validated using a different sample of CVD patients, aimed to construct a valid scale for assessing TMs related to lifestyle change. The survey statements were reframed to make them applicable for the current study (e.g., “My heart attack has made me realize that a healthy lifestyle is important to me” was reframed as “The corona crisis has made me realize that a healthy lifestyle is important to me”). Exploratory factor analyses and reliability analyses (results presented in Supplementary Material A) on the current data led to the final scales presented in Supplementary Material B. We separated intentions to change health behaviors, due to the COVID-19 crisis (i.e., TMs), by assessing four-item subscales related to TM for general lifestyle (α = 0.84), dietary behavior (α = 0.89), physical activity (α = 0.78), and smoking (α = 0.79), and a three-item subscale related to TM for alcohol consumption (α = 0.74). A mean score was calculated per health behavior, with higher scores indicating a higher intention to optimize a health behavior as a result of the COVID-19 crisis. Indicating, a higher perceived TM. All survey statements were answered on a seven-point Likert scale ranging from 1 = “strongly disagree” to 7 = “strongly agree.” These response options were selected as they generally demonstrate the most equal conceptual distances between the anchor labels (Casper et al., 2020).

As predictor variables, three components of the TM framework were employed, (1) risk perception, (2) affective impact, and (3) altered self-concept (McBride et al., 2003). In the absence of validated questionnaires specifically targeting these components during the COVID-19 pandemic, we included self-generated items with answer categories ranging from 1 = “not at all” to 7 = “totally.” Risk perception was assessed using four items targeting perceived risk of adverse health outcomes when infected with the COVID-19 virus (e.g., “Do you expect the coronavirus to have a worse effect on you than others of your age and gender?”). The four items were averaged to create a mean score (α = 0.83). Affective impact of the COVID-19 crisis was assessed using eight items targeting worry, stress, and negative emotions due to the outbreak (e.g., “Does the threat of the coronavirus make you anxious?”). The Cronbach's alpha for the total scale showed a good internal consistency in the sample (α = 0.83). After the removal of three items, this alpha increased to 0.88. The mean score of the resulting seven items was largely correlated (*r* = 0.67; *p* = < 0.01) with the Negative Affect Scale of the Positive and Negative Affect short form (PANAS-SF) (Thompson, 2007), which further validates the use of the self-generated items. Lastly, to assess self-concept, event-related measures are typically preferred due the changing nature of people's self that varies over the life course, particularly following significant life events (Demo, 1992; McBride et al., 2003). Self-concept can be defined as a perception about oneself or one's position in the grand scheme of things (Bergner and Holmes, 2000). As such, changed self-concept as a result of the COVID-19 crisis was assessed by two self-generated items with a Spearman-Brown correlation of 0.69 (i.e., “Has the corona crisis changed who you are as a person?” and “Has the corona crisis changed your outlook on life?”).

To assess the sample's sociodemographic characteristics, respondents were asked to report their age, gender, four-digit postal code, living situation (living alone or cohabitating), education, and employment status. Postal codes were linked to the Dutch Livability Index to score the livability of postal codes from 1 = “very insufficient' to 9 = “excellent” (Leidelmeijer et al., 2017). Level of education was classified according to the International Standard Classification of Education (ISCED) 2011 into lower education into lower education (none, elementary or vocational education), middle education (higher general and secondary vocational education), or higher education (higher professional and scientific education).

### Sample Size

We aimed to obtain a broad insight into lifestyle change intentions of Dutch CVD patients. In previous studies that investigated the role of risk perception, affective impact, and changed self-concept on behavior outcomes, a simple size of 218 and 59 was found to be underpowered to detect moderate or high effect sizes (McBride et al., 2017; Okely et al., 2019). This current study therefore aimed to recruit as many CVD patients as possible, and based the sample size on the expected availability and response rate of participants.

A total of 2,606 members of the patient association received the e-mail with an invitation to participate the questionnaire. From a total of 964 unique subjects who opened the link to the survey, 854 participants managed to complete all items (89% completion rate). Data from 24 participants had to be removed as they were not diagnosed with CVD (based on their answers on a question about CVD history), bringing the final sample size of our analyzed cohort to 830 participants.

### Statistical Analysis

We calculated and presented descriptive statistics (medians and frequencies) for sociodemographic characteristics, predictor and outcome variables. In addition, the percentages of participants that reported increased intentions to change a specific health behavior, due to COVID-19 crisis, were calculated using the box-score method. This box-score is the sum of percentages of the three most positive answer options on a 7-point Likert scale. Hence, participants with an averaged mean score of >5 (slightly agree) on the different outcome scales (Likert options 1 = strongly disagree, 2 = disagree, 3 = slightly disagree, 4 = neutral, 5 = slightly agree, 6 = agree, 7 = strongly agree) were denoted as participants with increased change intentions. An averaged mean score of ≥5 indicates that a participant demonstrates on average a slight intention to change a health behavior. This approach to determine favorable outcomes on Likert scales is in line with previous studies (Chipchase et al., 2012; Lapadula et al., 2021). Prior to performing regression analyses, we tested whether the variables met the assumptions of regression methodologies, including normally distributed residuals, linearity, and multicollinearity [based on VIF and correlations between predictors of <0.7 (Vatcheva et al., 2016)]. Linear regression analyses were carried out to examine the association between our three predictors (risk perception, affective impact, and altered self-concept) and the five TM outcome variables (increased intentions to change general lifestyle, physical activity, dietary behavior, alcohol consumption, and smoking). We first assessed the univariate association between each of our predictors and outcome variables. Subsequently, we ran two linear regression models for each of the TM outcome variable, using an enter selection strategy imputing the predictors. The three predictors specified above were entered in the first multivariate model. In the second model, we additionally adjusted for age, gender, living situation (living alone or cohabited), education (lower, middle, high), and presence of COVID-19 related symptoms. A *p*-value of 0.05 was considered significant. Statistical analyses were carried out using SPSS (version 25; IBM; Armonk, NY).

We conducted *post-hoc* sensitivity analyses to explore the robustness of our findings (Thabane et al., 2013). Firstly, we checked whether our findings differ when we change the outcome variables to single items that measure solely self-reported improved behavior (i.e., “Due to the corona crisis, I live healthier”). To do so, we repeated our regression analyses with these single items as outcome variables, rather than the complete TM scales. Secondly, the Extended Parallel Process model (EPPM) states that while some level of fear could induce behavioral change in order to reduce a certain threat, the highest level of fear may also lead to maladaptive behavior aimed at coping with fear itself (Witte, 1994). We therefore checked whether individuals who are particularly worried or emotional similarly affect our results. Hence, we repeated the regression analyses with only subjects scoring below the 75th percentile on the affective response scale.

## Results

### Sample Characteristics

The sociodemographic and disease characteristics of our final sample comprising 830 cardiovascular disease patients are presented in Table 1. Participants had a median age of 59 years (interquartile range (IQR) 52–65), and the majority of the sample was male (60%), cohabited with a partner (73%), completed higher education (49%), was currently unemployed or retired (77%), lived in a neighborhood with a (more than) good (62%) or (more than) adequate (30%) livability score (Leidelmeijer et al., 2017), and was diagnosed with coronary heart disease.

**Table 1:** Sociodemographic and disease characteristics of the sample (N = 830).

**Characteristic**	**Median (IQR)**	
**Age**	59 (52–65)	
	**Frequency (N)**	**Percentage (%)**
**Gender**		
Female/male	329/499	40/60
**Living situation**		
Living alone/cohabiting	213/604	25/73
**Education**		
Low/middle/high	161/259/406	20/31/49
**Employment**		
Employed	188	23
Unemployed	635	77
**Neighborhood livability**		
2 = more than insufficient	1	0.1
3 = insufficient	5	0.6
4 = weak	36	4
5 = adequate	31	4
6 = more than adequate	214	26
7 = good	260	31
8 = very good	154	19
9 = excellent	103	12
**Type of CVD**		
Coronary heart disease	591	71
Valvular heart disease	149	18
Cardiac arrhythmia	284	34
Other	407	48
**Presence of COVID-19 symptoms**		
Yes/no	187/831	23/77
**Current smokers**	64	8
**Alcohol consumption**	549	66

*Total percentages can deviate from 100% due to rounded numbers and/or multiple CVD problems*.

### Influence of the COVID-19 Crisis on Healthy Lifestyle Intentions

**Figure 2:**
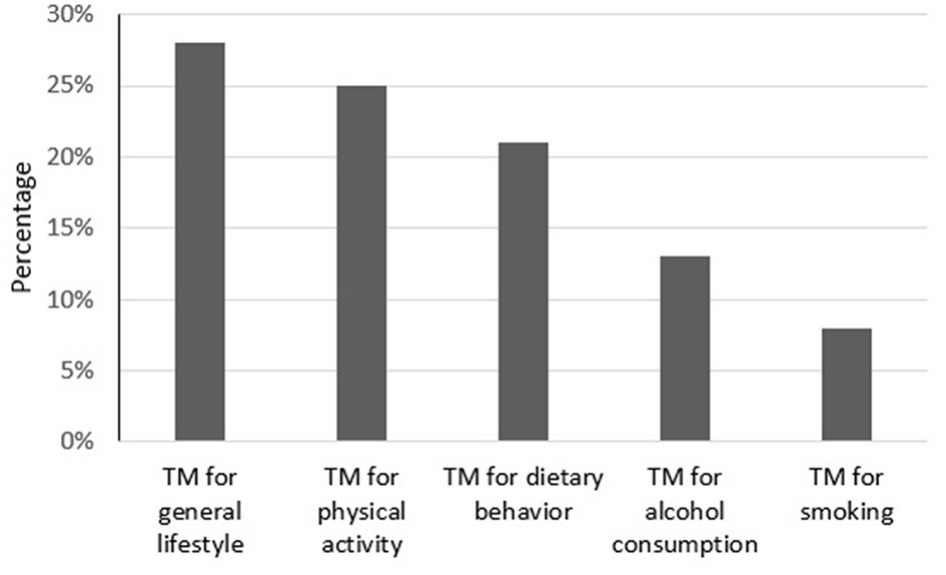
Averaged mean score of ≥5 on TM outcome variables.

The study initially explored the proportion of participants that expressed an increased intention to optimize lifestyle behaviors or noted increased importance of adopting a healthier lifestyle due to the COVID-19 crisis. Median scores and IQRs for predictor and outcome variables (range 1–7) are presented in Table 2. Figure 2 additionally provides sample percentages which indicate this increased interest in improving lifestyle or health behaviors due to the crisis (i.e., an averaged mean score of ≥5 indicating “slightly agree” on a Likert scale ranging from 1 = lowest intention to 7 = highest intention to improve lifestyle due to COVID-19). As seen in this figure, around a quarter of participants had an interest in becoming more physically active (25%) and in healthier eating (21%). A lower percentage of participants expressed an interest in changing smoking (8%) and alcohol habits (13%).

**Table 2:** Median (IQR) and range of TM outcome variables and predictors.

**Characteristic**	**Median (IQR)**	**Range**	**N**
**Outcome variables**			
TM			
Intention to change general lifestyle	4.1 (3.5–5.0)	1–7	822
Intention to change physical activity	4.0 (3.3–4.8)	1–7	819
Intention to change diet	4.0 (2.7–4.5)	1–7	814
Intention to reduce smoking	3.5 (2.0–4.0)	1–7	64
Intention to reduce alcohol	3.7 (2.7–4.3)	1–7	545
**Predictor variables**			
Risk perception	5.0 (4.3–6.0)	1–7	633
Affective response	3.3 (2.5–4.3)	1–7	723
Changed self-concept	3.5 (2.5–4.5)	1–7	827

### Predictors of TM for Changing Health Behaviors

The assumptions for regression analyses were checked, including linearity, normally distributed residuals and multicollinearity. No violations of these assumptions were found, thus indicating normally distributed residuals and linearity between the predictors and outcome variables. In addition, Table 3 provides the Pearson's correlation between our predictors. As all correlations are lower than 0.7 (Vatcheva et al., 2016), there was no evidence of multicollinearity.

**Table 3:** Pearson's correlation between predictors.

**Variables**	**Risk** **perception**	**Affective** **impact**	**Changed** **self-concept**
Risk perception	1		
Affective impact	0.32^*^	1	
Changed self-concept	0.11^*^	0.49^*^	1

* *p = < 0.01*.

Table 4 shows the results of the univariate and multiple linear regression analyses. A higher affective response and a greater change in self-concept were significantly associated with increased TM for general lifestyle. A higher risk perception was not significantly associated with an increased intention to change general lifestyle. The multivariate analysis revealed that only changed self-concept emerged as a significant predictor of TM for general lifestyle change (*B* = 0.26; CI = 0.19–0.33) after controlling for age, gender, living situation, education, and COVID-19 related symptoms. Similar results were found for TM for increasing physical activity. Again, only affective response and changed self-concept were significantly positively associated with TM for increasing physical activity in the univariate analyses, whilst solely changed self-concept emerged as a significant predictor in the multivariate model (*B* = 0.23; CI = 0.16–0.30). Higher risk perception, higher affective response and greater changes to self-concept were all associated with increased TMs for optimizing dietary behavior and lowering alcohol consumption. When analyzed in the multivariate model, both affective response (*B* = 0.12; CI = 0.04 – 0.21) and changed self-concept (*B* = 0.29; CI = 0.21–0.36) significantly predicted a TM for dietary behavior, after adjustment for covariates. Risk perception no longer significantly predicted TM for dietary behavior in the multivariate model. Similar predictors were found for a TM for lowering alcohol consumption; affective response (*B* = 0.11; CI = 0.01–0.20) and changed self-concept (*B* = 0.22; CI = 0.13–0.30). Finally, only changed self-concept was positively associated with a TM for smoking cessation, which was retained in the multivariate model (*B* = 0.29; CI = 0.01–0.57).

**Table 4:** Univariate and multivariate regression analyses.

	**Univariate** **analysis B**	**95% CI**	**Multivariate** **model 1 B**	**95% CI**	**Multivariate** **model 2 B**	**95% CI**
**TM for general lifestyle (*****N*** **=** **796)**			*R*^2^ = 0.10		*R*^2^ = 0.11	
Risk perception	0.05	−0.03, 0.12	0.00	−0.08–0.07	−0.02	−0.10–0.05
Affective response	0.20^***^	0.13, 0.27	0.06	−0.02–0.14	0.07^*^	−0.01–0.16
Changed self-concept	0.29^***^	0.23, 0.35	0.26^***^	0.19–0.33	0.26^***^	0.19–0.33
**TM for physical activity (*****N*** **=** **796)**			*R*^2^ = 0.08		*R*^2^ = 0.09	
Risk perception	0.06^*^	−0.01, 0.14	0.02	−0.05–0.10	0.01	−0.06–0.08
Affective response	0.17^***^	0.11, 0.24	0.04	−0.04–0.12	0.06	−0.03–0.14
Changed self-concept	0.26^***^	0.20, 0.32	0.24^***^	0.17–0.31	0.23^***^	0.16–0.30
**TM for dietary behavior (*****N*** **=** **794)**			*R*^2^ = 0.13		*R*^2^ = 0.14	
Risk perception	0.09^**^	0.01, 0.17	0.01	−0.06–0.09	0.00	−0.08–0.08
Affective response	0.29^***^	0.21, 0.36	0.13^***^	0.04–0.21	0.12^***^	0.04–0.21
Changed self-concept	0.35^***^	0.29, 0.42	0.29^***^	0.22–0.37	0.29^***^	0.21–0.36
**TM for alcohol consumption (*****N*** **=** **534)**			*R*^2^ = 0.10		*R*^2^ = 0.15	
Risk perception	0.13^***^	0.04, 0.22	0.08^*^	−0.01–0.16	0.04	−0.05–0.13
Affective response	0.24^***^	0.15, 0.32	0.08	−0.02–0.17	0.11^**^	0.01–0.20
Changed self-concept	0.28^***^	0.21, 0.36	0.23^***^	0.15–0.32	0.22^***^	0.13–0.30
**TM for smoking (*****N*** **=** **61)**			*R*^2^ = 0.14		*R*^2^ = 0.21	
Risk perception	0.21^*^	−0.04, 0.47	0.18	−0.11–0.47	0.20	−0.09–0.50
Affective response	0.23^*^	−0.01, 0.46	0.05	−0.27–0.36	0.03	−0.29–0.36
Changed self-concept	0.29^***^	0.08, 0.50	0.29^**^	0.01–0.56	0.29^**^	0.01–0.57

*Model 1, multivariate analysis with three predictors; model 2, multivariate analysis with three predictors, additionally adjusted for age, gender, living situation, education, and presence of COVID-19 symptoms.*

* *p < 0.10,*

** *p < 0.05,*

*** *p < 0.01*.

### Sensitivity Analyses

The regression analyses were repeated using single-item measures assessing a self-reported change in lifestyle, physical activity, dietary behavior, alcohol consumption, and smoking, due to COVID-19, as outcome variables rather than the multi-item TM scales. Our results were largely similar in terms of coefficients compared to the original analyses (data not shown). At the second sensitivity analysis where participants with responses above the 75th percentile on the affective response scale were excluded, comparable coefficients were found in the regression analyses (data not shown), suggesting patients most mentally affected by the COVID-19 crisis did not exude bias in the overall results.

## Discussion

We investigated the extent to which the COVID-19 crisis served as a teachable moment (TM) for changing health behavior among Dutch individuals living with cardiovascular disease (CVD), a disease responsible for 25% of the mortality in the Netherlands. Between 8 and 28% of the sample reported that they had optimized health behaviors or became more motivated to do so, due to COVID-19. Changes particularly related to general lifestyle (28%), physical activity (25%), and dietary behavior (21%), and to a lesser extent to limiting alcohol consumption (13%) and smoking (8%). These findings support our hypothesis that the COVID-19 pandemic may represent a TM regarding lifestyle change for a part of our sample; the outbreak seems to prompt some CVD patients to adopt risk-reducing health behaviors or makes them aware of the importance of doing so. We further explored the underlying motivation for behavior change intentions by investigating whether higher risk perception, affective impact, and changed self-concept were associated to the occurrence of a TM. These factors were derived from a theoretical framework (McBride et al., 2003), yet empirical evidence regarding its application to life events was still limited. We did not find evidence for the applicability of the whole TM framework. The extent to which the COVID-19 crisis was experienced as a TM was predominantly explained by an altered self-concept, resulting from the pandemic. This factor appeared to be the strongest predictor of intentions to change all health behaviors. An affective response toward the crisis did also have a facilitating role in the extent to which the crisis evoked a TM. CVD patients that were more affectively impacted by the COVID-19 crisis demonstrated increased intentions to adopt a healthier diet and to limit their alcohol consumption. Evidence for an important role of risk perception on evoking a TM was not found. CVD patients who perceived themselves to be more at risk for adverse health outcomes during a COVID-19 infection did not demonstrate higher intentions to adopt a healthier lifestyle.

### Comparison With Other Studies

Other studies that have investigated lifestyle during the COVID-19 pandemic have shown varying outcomes. Mainly reporting improved dietary behaviors but also decreased physical activity levels, largely due to an increase in sedentary behavior (Chopra et al., 2020; Hu et al., 2020; Knell et al., 2020). Consistent with previous studies, approximately one fifth of our sample reported increased motivation to improve their diet due to the pandemic. These findings add to the evidence base suggesting significant life events such as a disease diagnosis or becoming a parent are associated with improved eating behaviors (Polhuis et al., 2020). In contrast to previous studies (Chopra et al., 2020; Hu et al., 2020; Knell et al., 2020), the best-represented TM in our study related to improving physical activity, with a quarter expressing greater motivation to increase their activity. In our sample, 8% of the smokers were more motivated to quit smoking due to the pandemic, a figure slightly below other health behaviors. Although these results are similar to another study on the influence of the corona crisis on smoking habits (Bommele et al., 2020), we expected this figure to be higher, in light of increasing evidence emphasizing the hazards of smoking for the respiratory COVID-19 disease (Vardavas and Nikitara, 2020). Caution when interpreting our results is warranted, as our sample only included 8% smokers compared to 21% in the general adult Dutch population (Volksgezondheidenzorg.info, 2020), probably due to recommendations that CVD patients should avoid smoking. Smokers who continued to smoke following a diagnosis of CVD may not adequately perceive quitting smoking as important or may experience extreme difficulty with smoking cessation. Moreover, it is possible that patients have more opportunity to smoke, because of home-isolation, or experience stress, which in turn may increase tobacco use (Slopen et al., 2013).

### Explanation of the Findings

The role of the three factors of the TM framework (McBride et al., 2003) on behavior change intentions can be explained by existing psychological theories reported in previous studies on health behavior. First, our results confirm an association between a TM and a change in self-concept; the perception of oneself or one's position in life (Bergner and Holmes, 2000). The more individuals indicated that the corona crisis changed their sense of self, who they are as a person or their outlook on life, the more they perceived the outbreak as a turning point toward improving lifestyle. An explanation for this phenomenon can be found in identity theories (Kearney and O'Sullivan, 2003). Significant life events can cause an identity shift, prompting people to become more self-aware and thereby to re-evaluate current health behaviors and their conflicting effect on future health goals (Kearney and O'Sullivan, 2003). A transition in self-concept or identity thus facilitates behavior change (Meijer et al., 2020). Events that endanger positive expectations about their future self are usually experienced as most relevant to individuals, which in turn evoke greater behavioral responses (Aspinwall and Brunhart, 1996; McBride et al., 2003). In an extensive qualitative study, it was explained that major life events impacted an individual's outlook on life and thereby made them increasingly thoughtful regarding the effects of their current diet (Polhuis et al., 2020). Our finding that a higher level of altered self-concept was associated with increased intentions toward behavioral change provides evidence that the COVID-19 crisis induces a comparable effect.

Second, our results confirm that an affective response toward the COVID-19 crisis was associated with the occurrence of a TM for improving nutrition and reducing alcohol consumption. This association is in line with existing research on affect and health behaviors, that emphasizes the relation between emotions and beneficial behavior-related decision making (Lerner et al., 2015). Emotionally laden contexts or events could have a cueing effect on deciding to adopt protective health behaviors (DeSteno et al., 2013; Williams et al., 2019). According to the framework described by McBride et al. (2003), an emotional response is essential for an event to become a TM. This affective response causes events to be perceived as more meaningful and significant. Negative emotions such as worry and fear raise concern about health problems and increase motivation to eliminate health risks by adopting risk-reducing behaviors (McBride et al., 2008). In a study conducted by Knell et al. (2020), health concerns were also reported as the main reason for avoiding negative health behaviors during the pandemic, in particular related to a reduction in alcohol consumption and smoking.

Third, although perceived risk of severe health outcomes when infected with the COVID-19 virus was associated with a TM for lifestyle change, this association was no longer significant in the complete regression models. While our sample, on average, perceived themselves to be highly susceptible, this perception was not associated with healthier lifestyle intentions. This is in line with a study by Vörös et al. (2018), who neither found a link between patient's estimations of their own cardiovascular risk and their willingness to change their lifestyle. According to the Health Belief Model (HBM) theory, the likelihood of engaging in health-protective behaviors is explained by both the perceived threat of a disease as well as perceptions regarding the effectiveness of behaviors adopted to counteract that threat (Becker, 1974). It is thus crucial that individuals believe that adopting risk-reducing health behaviors will actually reduce the health threat of COVID-19. Although we did not take behavior expectations into account in our study, it is possible that the relevance of lifestyle in relation to COVID-19 was unfamiliar to participants at the time of data collection. Future research should thus continue to explore the role of risk perception following significant life events, but specifically include perceived risk linked to unhealthy lifestyle. Moreover, the protective effects of engaging in a healthy lifestyle during the current pandemic should be clearly and empathically emphasized to CVD patients.

### Implications

The potential TM for CVD patients presents some considerable implications concerning how to capitalize on the corona crisis as a TM. During potential TMs, individuals have a heightened receptivity for health behavior messages (McBride et al., 2003; Lawson and Flocke, 2009; Cohen et al., 2011). Health authorities, (mental) healthcare professionals, patient organizations, and governments should therefore actively provide health behavior education during and after the current pandemic. In doing so, they might encourage CVD or other chronic patients to utilize the pandemic as a turning point toward a healthier lifestyle. Targeting risk perception is already a frequently used behavior change approach in health promotion interventions (Ferrer and Klein, 2015). Although this could be an effective strategy in some situations, our results imply that it may be valuable to additionally draw attention to an altered self-concept and affective responses in relation to an event. Specifically, by emphasizing the extent to which the pandemic has altered patients' self-image or their world image, patients may be redirected toward new health behaviors and habits that fit these new identity perceptions (Kearney and O'Sullivan, 2003; Caldwell et al., 2018). Further, motivation for lifestyle change may increase by acknowledging and targeting the affective impact of the COVID-19 crisis, and subsequently linking these to risk-reducing health behaviors (McBride et al., 2008).

### Strengths and Limitations

Our study had several noticeable strengths. First, we mention the large sample size and the diverse demographic characteristics of the population we approached. Second, to our knowledge this is the first study to investigate the influence of the COVID-19 crisis on the lifestyle attitudes of CVD patients. Our results provide important guidelines that inform behavioral guidance programs during current and future public health threats. The third strength we experienced, was the use of measures to assess the TM outcome variables based on high construct validity, and the use of specific event-related measures to assess risk perception, affective response and changed self-concept, as advocated by McBride et al. (2003). Lastly, individuals with cardiometabolic disorders are part of the largest chronic disease patient group worldwide (World Health Organization, 2020b). Our findings could therefore substantially benefit a large number of individuals globally.

However, future research studies should also take certain limitations into account. First, although investigating a single patient population typically enhances internal validity of findings, our focus on CVD patients alone is likely to reduce the generalizability of our findings to other patient population or to the general population (Kukull and Ganguli, 2012). Less vulnerable groups could experience a lesser sense of urgency to adopt risk-reducing health behaviors, and are thus possibly less likely to perceive the COVID-19 crisis as a TM. However, we expect that the extent of changed self-concept and affective impact of the crisis could explain and predict behavior change intentions of other patient groups as well. Further research is necessary to confirm this assumption. Second, since the majority of our participants was member of a CVD patient association, our sample might have a higher-than-average interest in employing a healthy lifestyle, which could affect the generalizability of our results. Also, although we aimed to approach CVD patients with diverse sociodemographic characteristics, nearly half of our participants completed higher education. This overrepresentation of higher educated people is a common phenomenon in science, presumably because this group has a greater interest in health and science (Enzenbach et al., 2019). Yet, as our sample also consisted of a reasonable percentage of lower-educated participants and those with unhealthier lifestyle behaviors (e.g., smokers), bias on the observed associations may be likely toward the null. Third, the use of self-generated scales may impact the reproducibility of our results, thus further longitudinal data collection is needed. Fourth, given the self-report design of the study, lifestyle behaviors could not be validated on the basis of objective measures such as pedometers and a risk of recall bias cannot therefore be entirely discounted. Moreover, the use of a cross-sectional design prevented us from exploring lifestyle changes during COVID-19 over longer time periods. Lastly, although the study was actually based on a valid conceptual model, determinants of other behavior change theories may have also been worth exploring. Future studies should expand the study design with additional behavior change determinants, including perceived risk linked to unhealthy lifestyle.

## Conclusion

In summary, the COVID-19 crisis may present a teachable moment for CVD patients, making them increasingly motivated to change health behaviors and increasingly receptive for behavioral messages. A change in a person's self-concept, due to the COVID-19 crisis, and experiencing an affective response toward the crisis, were both associated with increased intentions to change lifestyle behaviors. These results highlight a window of opportunity for healthcare professionals to utilize the pandemic to promote a healthy lifestyle among their patients. Employing targeted lifestyle advice during or shortly after the current COVID-19 crisis may encourage CVD patients to bring about desired behavioral changes, with a relatively modest effort.

## Data Availability Statement

The raw data supporting the conclusions of this article will be made available by the authors upon request, without undue reservation.

## Ethics Statement

The studies involving human participants were reviewed and approved by The Medical Ethics Committee of Leiden University Medical Center. The patients/participants provided their written informed consent to participate in this study.

## Author Contributions

MB and JK contributed to the conception or design of the study. WG, MN, and JK supervised the overall study. MB, WG, and JK contributed to data analysis and interpretation of data. MB drafted the manuscript in close collaboration with WG and JK. All authors critically revised the manuscript, gave final approval and agreed to be accountable for all aspects or work, ensuring integrity, and accuracy.

## Conflict of Interest

The authors declare that the research was conducted in the absence of any commercial or financial relationships that could be construed as a potential conflict of interest.
