# Supplementary material for: The COVID-19 Crisis as a Teachable Moment for Lifestyle Change in Dutch Cardiovascular Disease Patients
Source: Front Psychol. 2021 Jun 22;12:678513. doi: 10.3389/fpsyg.2021.678513 (PMC8259785; doi:10.3389/fpsyg.2021.678513)
Supplement: Supplementary file 1 [file Table_1.DOCX]

Supplementary Material

Exploratory factor analyses and reliability analyses on outcome variables

| **Table 1.**  Rotated Factor Matrix and reliability analysis TM for general lifestyle | | | | | | | |
| --- | --- | --- | --- | --- | --- | --- | --- |
| **Item** | **2-factor structure ^a, b^** | |  | **1-factor structure** |  | **Corrected item-total correlation** | **Cronbach's alpha if item deleted** |
|  | **1** | **2** |  | **1** |  |  |  |
| 1. Due to the corona crisis, I more strongly feel the necessity of adopting a healthy lifestyle. | 0.73 | 0.07 |  | 0.73 |  | 0.63 | 0.64 |
| 2. Due to the corona crisis, I allow myself more time to pursue a healthy lifestyle. | 0.78 | -0.19 |  | 0.75 |  | 0.60 | 0.65 |
| 3. The corona crisis has made me realize that a healthy lifestyle is important to me. | 0.76 | 0.12 |  | 0.77 |  | 0.65 | 0.63 |
| 4. For me, my lifestyle is fine the way it is (reversed). | 0.02 | 0.34 |  | 0.03 |  | 0.30 | **0.84 ^c^** |
| 5. Due to the corona crisis, I live healthier. | 0.76 | 0.12 |  | 0.76 |  | 0.66 | 0.63 |
| **R^2^ factors** | 45.74% | 3.65% |  |  |  |  |  |
| **R^2^ total** | 49.39% | |  | 45.36% |  |  |  |
| Extraction Method: Principal Axis Factoring. Rotation Method: Varimax with Kaiser Normalization.  a. Rotation converged in 3 iterations.  b. The second factor accounted only for an additional 3.65%, therefore 1-factor structure more appropriate.  c. Item 4 will be deleted. | | | | | | | |

| **Table 2.** Factor Matrix and reliability analysis TM for physical activity | | | | |
| --- | --- | --- | --- | --- |
| **Item** | **1-factor structure ^a^** |  | **Corrected item-total correlation** | **Cronbach’s alpha if item deleted** |
| 1. Due to the corona crisis, I more strongly feel the necessity of sufficient physical activity. | 0.62 |  | 0.63 | 0.41 |
| 2. Due to the corona crisis, I allow myself more time to exercise. | 0.80 |  | 0.45 | 0.51 |
| 3. The corona crisis has made me realize that sufficient physical activity is important to me. | 0.59 |  | 0.62 | 0.41 |
| 4. For me, my amount of physical activity is fine the way it is (reversed). | 0.17 |  | 0.11 | **0.78 ^b^** |
| 5. Due to the corona crisis, I exercise more. | 0.72 |  | 0.39 | 0.54 |
| **R^2^ total** | 38.59% |  |  |  |
| Extraction Method: Principal Axis Factoring. a. 10 iterations required.  b. Item 4 will be deleted. | | | | |

| **Table 3.** Factor Matrix and reliability analysis TM for dietary behavior | | | | |
| --- | --- | --- | --- | --- |
| **Item** | **1-factor structure ^a^** |  | **Corrected item-total correlation** | **Cronbach’s alpha if item deleted** |
| 1. Due to the corona crisis, I more strongly feel the necessity of adopting a healthy diet. | 0.83 |  | 0.76 | 0.72 |
| 2. Due to the corona crisis, I allow myself more time to prepare and consume healthy meals. | 0.78 |  | 0.67 | 0.75 |
| 3. The corona crisis has made me realize that a healthy/healthier diet is important to me. | 0.86 |  | 0.77 | 0.72 |
| 4. For me, my diet is fine the way it is (reversed). | 0.11 |  | 0.11 | **0.89 ^b^** |
| 5. Due to the corona crisis, I eat healthier. | 0.81 |  | 0.72 | 0.72 |
| **R^2^ total** | 54.16% |  |  |  |
| Extraction Method: Principal Axis Factoring. a. 6 iterations required.  b. Item 4 will be deleted. | | | | |

| **Table 4.** Rotated Factor Matrix and reliability analysis TM for lowering alcohol consumption | | | | | | | |
| --- | --- | --- | --- | --- | --- | --- | --- |
| **Item** | **2-factor structure ^a, b^** | |  | **1-factor structure ^c^** |  | **Corrected item-total correlation** | **Cronbach's alpha if item deleted** |
|  | **1** | **2** |  | **1** |  |  |  |
| 1. Due to the corona crisis, I more strongly feel the necessity of sticking to this advice of the Dutch Heart Foundation. | 0.83 | 0.04 |  | 0.85 |  | 0.57 | 0.28 |
| 2. The corona crisis has made me realize that sticking to this advice of the Dutch Heart Foundation is important to me. | 0.81 | 0.10 |  | 0.80 |  | 0.57 | 0.29 |
| 3. For me, my alcohol consumption is fine the way it is (reversed). | 0.00 | 0.25 |  | 0.01 |  | 0.01 | **0.74 ^d^** |
| 4. Due to the corona crisis, I consume less alcohol. | 0.48 | -0.22 |  | 0.46 |  | 0.34 | 0.50 |
| **R^2^ factors** | 39.41% | 3.06% |  |  |  |  |  |
| **R^2^ total** | 42.47% | |  | 39.20% |  |  |  |
| Extraction Method: Principal Axis Factoring. Rotation Method: Varimax with Kaiser Normalization.  a. Rotation converged in 2 iterations.  b. The second factor accounted only for an additional 3.06%, therefore 1-factor structure more appropriate.  c. 15 iterations required  d. Item 4 will be deleted. | | | | | | | |

| **Table 5.** Factor Matrix and reliability analysis TM for smoking cessation | | | | |
| --- | --- | --- | --- | --- |
| **Item** | **1-factor structure ^a^** |  | **Corrected item-total correlation** | **Cronbach’s alpha if item deleted** |
| 1. Due to the corona crisis, I more strongly feel the necessity of quitting smoking. | 0.87 |  | 0.73 | 0.66 |
| 2. The corona crisis has made me quit smoking. | 0.56 |  | 0.50 | 0.75 |
| 3. The corona crisis has made me realize that quitting smoking is important to me. | 0.87 |  | 0.74 | 0.66 |
| 4. For me, my smoking behavior is fine the way it is (reversed). | 0.44 |  | 0.39 | **0.79 ^b^** |
| 5. The corona crisis has made me smoke less. | 0.50 |  | 0.43 | 0.77 |
| **R^2^ total** | 45.52% |  |  |  |
| Extraction Method: Principal Axis Factoring. a. 7 iterations required.  b. Item 4 will be deleted. | | | | |
