# Supplementary material for: The COVID-19 Crisis as a Teachable Moment for Lifestyle Change in Dutch Cardiovascular Disease Patients
Source: Front Psychol. 2021 Jun 22;12:678513. doi: 10.3389/fpsyg.2021.678513 (PMC8259785; doi:10.3389/fpsyg.2021.678513)
Supplement: Supplementary file 2 [file Table_2.DOCX]

Supplementary Material B

Final survey

**TM for improving general lifestyle:**

|  | Strongly disagree | Disagree | Somewhat disagree | Neutral | Somewhat agree | Agree | Strongly agree |
| --- | --- | --- | --- | --- | --- | --- | --- |
| 1. Due to the corona crisis, I more strongly feel the necessity of adopting a healthy lifestyle. |  |  |  |  |  |  |  |
| 2. Due to the corona crisis, I allow myself more time to pursue a healthy lifestyle. |  |  |  |  |  |  |  |
| 3. The corona crisis has made me realize that a healthy lifestyle is important to me. |  |  |  |  |  |  |  |
| 4. Due to the corona crisis, I live healthier. |  |  |  |  |  |  |  |

**TM for physical activity:**

|  | Strongly disagree | Disagree | Somewhat disagree | Neutral | Somewhat agree | Agree | Strongly agree |
| --- | --- | --- | --- | --- | --- | --- | --- |
| 1. Due to the corona crisis, I more strongly feel the necessity of sufficient physical activity. |  |  |  |  |  |  |  |
| 2. Due to the corona crisis, I allow myself more time to exercise. |  |  |  |  |  |  |  |
| 3. The corona crisis has made me realize that sufficient physical activity is important to me. |  |  |  |  |  |  |  |
| 4. Due to the corona crisis, I exercise more. |  |  |  |  |  |  |  |

**TM for dietary behavior:**

|  | Strongly disagree | Disagree | Somewhat disagree | Neutral | Somewhat agree | Agree | Strongly agree |
| --- | --- | --- | --- | --- | --- | --- | --- |
| 1. Due to the corona crisis, I more strongly feel the necessity of adopting a healthy diet. |  |  |  |  |  |  |  |
| 2. Due to the corona crisis, I allow myself more time to prepare and consume healthy meals. |  |  |  |  |  |  |  |
| 3. The corona crisis has made me realize that a healthy/healthier diet is important to me. |  |  |  |  |  |  |  |
| 4. Due to the corona crisis, I eat healthier. |  |  |  |  |  |  |  |

**TM for lowering alcohol consumption:**

*The general advice of the Dutch Heart Foundation concerning alcohol consumption is: do not drink alcohol or drink a maximum of 1 glass of alcohol per day.*

|  | Strongly disagree | Disagree | Somewhat disagree | Neutral | Somewhat agree | Agree | Strongly agree |
| --- | --- | --- | --- | --- | --- | --- | --- |
| 1. Due to the corona crisis, I more strongly feel the necessity of sticking to this advice of the Dutch Heart Foundation. |  |  |  |  |  |  |  |
| 2. The corona crisis has made me realize that sticking to this advice of the Dutch Heart Foundation is important to me. |  |  |  |  |  |  |  |
| 3. Due to the corona crisis, I consume less alcohol. |  |  |  |  |  |  |  |

**TM for smoking cessation:**

|  | Strongly disagree | Disagree | Somewhat disagree | Neutral | Somewhat agree | Agree | Strongly agree |
| --- | --- | --- | --- | --- | --- | --- | --- |
| 1. Due to the corona crisis, I more strongly feel the necessity of quitting smoking |  |  |  |  |  |  |  |
| 2. Due to the corona crisis, I smoke less. |  |  |  |  |  |  |  |
| 3. The corona crisis has made me realize that quitting smoking is important to me. |  |  |  |  |  |  |  |
| 4. The corona crisis has made me quit smoking. |  |  |  |  |  |  |  |

**Risk perception:**

|  | Not at all | No | Not really | Neutral | A little | Yes | Absolutely |
| --- | --- | --- | --- | --- | --- | --- | --- |
| 1. Do you belong to a risk group for adverse complications of the coronavirus? |  |  |  |  |  |  |  |
| 2.Do you expect serious complications when you become infected with the coronavirus? |  |  |  |  |  |  |  |
| 3. Do you expect the coronavirus to have a worse effect on you than others of your age and gender? |  |  |  |  |  |  |  |
| 4. Do you expect to survive a coronavirus infection? |  |  |  |  |  |  |  |

**Affective response:**

|  | Not at all | No | Not really | Neutral | A little | Yes | Absolutely |
| --- | --- | --- | --- | --- | --- | --- | --- |
| 1. Are you concerned about the threat of the coronavirus? |  |  |  |  |  |  |  |
| 2. Does the threat of the coronavirus make you anxious? |  |  |  |  |  |  |  |
| 3. Does the threat of the coronavirus make you gloomy? |  |  |  |  |  |  |  |
| 4. Are you experiencing stress due to the corona crisis? |  |  |  |  |  |  |  |
| 5. Are you sleeping worse due to the corona crisis? |  |  |  |  |  |  |  |
| 6. Do you suffer from nightmares about the corona crisis? |  |  |  |  |  |  |  |

**Changed self-concept:**

|  | Not at all | No | Not really | Neutral | A little | Yes | Absolutely |
| --- | --- | --- | --- | --- | --- | --- | --- |
| 1. Has the corona crisis changed who you are as a person? |  |  |  |  |  |  |  |
| 2. Has the corona crisis changed your outlook on life? |  |  |  |  |  |  |  |
